# Implications of Unconnected Micro, Molecular, and Molar Level Research in Psychology: The Case of Executive Functions, Self-Regulation, and External Regulation

**DOI:** 10.3389/fpsyg.2019.01919

**Published:** 2019-08-27

**Authors:** Jesús de la Fuente, María Carmen González-Torres, Maite Aznárez-Sanado, José Manuel Martínez-Vicente, Francisco Javier Peralta-Sánchez, Manuel Mariano Vera

**Affiliations:** ^1^School of Education and Psychology, University of Navarra, Pamplona, Spain; ^2^School of Psychology, University of Almería, Almería, Spain; ^3^Center of Research of Psychology, University of Almería, Almería, Spain; ^4^School of Psychology, University of Granada, Granada, Spain

**Keywords:** research levels, psychology, executive functions, self-regulation, self-control, external regulation

## Abstract

The proliferation of research production in Psychology as a science has been increasing exponentially. This situation leads to the necessity of organizing the research production into different levels of analysis that make it possible to delimit each research domain. The objective of this analysis is to clearly distinguish the different levels of research: micro-analysis, molecular, and molar. Each level is presented, along with an analysis of its benefits and limitations. Next, this analysis is applied to the topics of *Executive Functions*, *Self-Regulation*, and *External Regulation*. Conclusions, limitations, and implications for future research are offered, with a view toward a better connection of research production across the different levels, and an allusion to ethical considerations.

## Introduction

With the recent proliferation of a large volume of research in Neuroscience, Cognitive Neuropsychology and Psychology, referring to the neurological substratum of basic psychological processes, we find an epistemic imbalance where the more global, contextualized approaches to explaining behavior give way to approaches based on analysis and study of basic processes. Based on observation of this reality, the aim of the following theoretical analysis is: (1) to define the different levels that make up psychological research, as well as the scientific domain inherent to each, with their limitations and benefits; and (2) to apply this classification to a recent research topic of great academic, investigative, and professional impact: executive functions and self-regulation.

The development of human knowledge in an *empirical scientific format*, as we know it today, is relatively recent in human history, given its complexity and the cognitive repertoires and high-level technology that are required to produce it. It is not surprising, therefore, that the initial formats of knowledge were conceptual constructions taken from related *facts*, which were used to develop *concepts* (definitions, characteristics), and finally, patterns or probabilistic predictions in the form of *principles.* Fables, proverbs and parables are good examples of this kind of commonplace psychological knowledge. All such elaborations have a common denominator: they start from direct observation of reality, based on unfiltered samples of behavior, from which they propose probabilistic relations and common-sense predictions. This type of understanding, therefore, is a form of unscientific, untested, folk knowledge.

With the historical appearance of the *scientific-positivist paradigm*, production of human knowledge in a scientific format has greatly increased, both in quality and in quantity. This production, however, has clearly been conditioned by the resources or technological developments available at the moment. Commonplace human knowledge thus tends to increasingly match contributions of what we call scientific knowledge. Consequently, in today’s *Knowledge Society* heavily laden with this type of knowledge and the scientific-technological component any analysis of reality or of any issue is usually connected to some type of evidence or some scientific-technical grounding. This does not mean that scientific knowledge is infallible or unquestionable (the overtrust bias); instead, such knowledge is subject to being *disproven, restricted* or *verified*, to ensure that it attains a higher level of consistency than folk knowledge, which is elaborated unsystematically by a human cognitive system, with many inaccuracies, inconsistencies and biases. On the other hand, scientific knowledge would have been obtained using technological instruments and techniques that provide for greater accuracy in data collection, processing, and analysis. This fact has led to a perception of data obtained through high level techniques as being “more scientific” (the technology bias). As a result, recent scientific studies involving complex techniques (scanners, computed tomography, etc.) would seem to have more scientific value. Psychology as a science is not invulnerable to this scenario. In this line, McCabe and Castel (2008) demonstrated that, in the area of cognition, the inclusion of brain images in an article results in increased scores for its scientific reasoning. In this case, the authors argue that this might be explained by “people’s affinity for reductionistic explanations of cognitive phenomena” (p. 343).

In light of this, it seems appropriate to reflect on the different levels and formats of psychology research – to clarify their different theoretical and empirical domains, and so encourage a better connection and integration between them, in both conceptual and applied knowledge. In order to illustrate these concepts, examples related to executive functions, their associated behavioral and contextual variables/models and their relationship with ADHD will be discussed.

## Levels and Formats of Psychology Research

### Definition of Levels and Formats

The different levels of research have coexisted within Science from the beginning, and they contribute complementary elements to the analysis of phenomena. In the case of Psychology, one may establish three levels of analysis of human behavior. To grasp this more clearly, we use the *journey metaphor* (Pintrich, 2000a, b) *adapted*. See Table 1.

**TABLE 1:** Levels of research and working formats in the “building” of Psychological Research.

**Metaphor**	**Level**	**Format**	**Type of variables**	**Techniques**	**Models**	**Implications**
(3) Journey	MOLAR (person in context)	Interactive and contextual/applied	Subject × context self-regulation externally-regulated.	Observation, self-reports other-reports experiments	SR vs. ER	Practices applied to molar and contextualized processes
(2) Car	MOLECULAR (person)	Discrete/conceptual personalistics	Subject (context) meta-attention meta-motivation self-control self-regulation	Observation self-reports experiments clinical cases	SR (or SRL)	Practices applied to molecular and molar processes
(1) Engine	MICRO-ANALYSIS (brain)	Basic/neurological	Brain, microprocesses medication	Tomography, scanner tests clinical cases	EF	Practices applied to basic and Molecular processes

*EFs, executive functions; SR, self-regulation; SRL, Self-Regulated Learning; SRL vs. ERL, Self-Regulated vs. Externally Regulated Learning.*

(1) Level of *micro-analysis* of behavior (biological analysis or the “hardware” of behavior). As the name indicates, it focuses on the smallest, most discrete, most basic level of the phenomenon being studied. In the case at hand, we may consider this level of analysis as referring to the biological, neuronal substrata or underpinnings of human conduct. Hence, the name neuropsychology, referring to the neuronal-behavioral connection. Typical of this domain of analysis is identifying areas of the brain, their connections, explanations, regularities, and biological or neuronal models of behavior. Consequently, this domain studies behavioral problems or disorders that stem from biological or physiological processes. One highly relevant, current line of research at this level is that of the neurological foundations of psychological processes and executive functions (Lukito et al., 2018; Rubia, 2018).
(2) Level of *molecular analysis* of behavior (functional analysis or the “software” of behavior). In this case, the analysis focuses on constructing *behavioral models* of the types of learning that are operational in human beings. Examples at this level would be theories, laws and models of learning – studied profusely and mastered by psychologists– in order to explain different types of human behavior, and thereby enable precise behavioral analyses of problems and behavioral disorders at the molecular level, or mid-level of complexity. These models also serve to promote and optimize processes of human development and learning. This is the level where we find models of self-regulation (Brown, 1998) and self-regulated learning (Zimmerman, 2000; Schunk, 2005).
(3) Level of *molar analysis* of behavior (interactive analysis or *analysis of behavior in real, interactive contexts*). Analysis at this level is necessarily interactive because the object of analysis is the subject-situation binomial, in interaction, in real contexts. At this level we find the contextualized, applied models of behavior, typical of the applied disciplines of Psychology, such as Educational Psychology, Social Psychology and Clinical and Health Psychology. These disciplines seek to explain, model and predict the influence of factors relating to the subject and to the situation, and to estimate the proportion of variance that is explained by the two groups of factors, in order to determine likely behaviors. In our specific topic area, it would be Level 3 of the theoretical model referred to as the “Theory of Self-Regulated vs. Externally-Regulated Learning,” or SRL vs. ERL Theory (de la Fuente, 2017).

### Issues Associated With the Different Levels of Research Inference

As we have seen, each level of research involves certain characteristics, benefits and limitations that should be made explicit in order to adequately contextualize research production. Lacking this, inferences may be drawn that are not appropriate to the particular domain of scientific production; in other words, data and models from one level may be used to try to explain phenomena beyond the scope or domain of the investigation in question, using high-level inferences. For this reason, it is valuable to recall the benefits and limitations inherent to each level of research.

#### Level of Micro-Analysis

As a *benefit*, this level ascertains precise information about the biochemical, psychophysiological, and neurological areas and processes of behavior. It is therefore essential for constructing models and information related to disorders and problems of a neuropsychological and neurophysiological nature. However, this detailed, discrete vision of behavior has the *limitation* that linear implications may not be extracted and applied to the molecular level, much less to the molar. As in the Table 1 metaphor, knowledge of how the car engine is wired does not help us understand any substantial variance of the car’s total behavior (the subject), or how the trip transpires along the different roads (context). It is obvious that a good engine increases the likelihood of a good car and a good trip, but there are many intervening (mediating) variables between the car engine and the car’s behavior on the road that need to be understood in order to have a complete view of the phenomenon being studied – such as the style of driving and the road conditions.

#### Molecular Analysis Level

This level of analysis has the *benefit* of ascertaining mid-range information and enabling the creation of mid-level explanatory models. Following the Table 1 metaphor, this level would provide knowledge about the car’s behavior, understood as the set of factors referring to and mediated by the subject who drives the car. In this case we are considering the driver’s manner of driving. It is obvious that a good engine increases the likelihood of a good driving style, but we understand that they are two different variables; they cannot be further reduced, since a good engine can be part of a car with other differentiating characteristics. A good engine with an inefficient braking system or downforce, associated with a reckless driving style, only increases the chances of a traffic accident. Nonetheless, this level of analysis also has *limitations* or restrictions in that it contributes no information about the characteristics of the context. Consequently, we need a further level of analysis in order to offer a contextualized, interactive view of the object of study.

#### Level of Molar Analysis

The *benefit* of this level of analysis lies in its effort to explain subjects’ behavior in interaction with characteristics of the real context, paying special attention to the causative role of the context in the behavior being analyzed. The *limitation* of this level is the difficulty of descending from here to connect with the micro-analysis level. In the Table 1 metaphor, this type of analysis would be equivalent to constructing models that explain and predict the role of the road (traffic signs, design, camber, course) in subjects’ behavior, and how subjects interact with such contextual characteristics.

Based on the above analysis, we can identify the most common research errors that result from not clearly establishing the contributions and limitations of the domain of one’s object of study.

#### Compartmentalization Error

This error refers to not clearly defining the connections, differences, and continuity between similar constructs, adopted at different levels. In practice, this results in research using different conceptual labels for similar or equivalent constructs that emerge at different research levels. For example, adopting labels such as executive functions in reference to the self-regulation construct without previously defining the models and the domain or theoretical level at which these appear leads to conceptual confusion between constructs and their levels of application. A visual illustration would be researchers working on three different floors of a building (micro-analysis, molecular analysis, molar analysis), all of whom are studying the behavior of a car, but unaware of what the researchers on the other floors are working on (see Table 1).

#### Overgeneralization Error

In this case research conclusions or consequences are inferred at a level or domain distant from the data or evidence provided. For example, since basic research on attentional difficulties has shown that smells contribute to learning, one might conclude that this variable ought to be included in teaching-learning processes at school. This is a high-level inference, given that this evidence can easily be applied and integrated into discrete, basic instrumental models of learning, basic learning processes through classic laboratory conditioning of human beings. However, it is much more complicated to apply this to cognitive, meta-cognitive and meta-motivational models that explain processes involved in the construction of complex knowledge, in real contexts of formal learning.

#### Fuzziness Error

This refers to defining conceptually similar constructs, taken from one’s own level of research or another, and using it to justify results at a different level of research. For example, to assume that inattention results directly and exclusively from dysregulation of executive functions, as a neurological construct, when the evidence has consistently shown that strategic processes are involved, including meta-attention and behavioral, emotional and cognitive dysregulation. As advise Lilienfeld et al. (2015), clarity and rigor are essential in disciplines as psychology and psychiatric where many of the phenomena studied are “open concepts” that favor misunderstanding.

## The Case of Executive Functions, Self-Regulation, and External Regulation

### Levels of Analysis

#### Micro-Analysis Level of the Problem (Behavioral “Hardware”): Executive Functions

##### Definition and typology

Executive functions refer to higher-order control processes involved in the regulation of thought and action. Barkley (1997a) defined executive functions as “those types of actions we perform to ourselves and direct at ourselves so as to accomplish self-control, goal-directed behavior, and the maximization of future outcomes” (p. 57). Historically, there has been great interest and proliferation of studies focused on determining the role of the frontal lobes in human behavior, where consideration of that role has evolved from negligent to being central to the explanation of self-regulated human behavior (Fuster, 1989, 1997, 2002; Casey, 1992; Barkley, 1997a, b; Elliot, 2003; Eshel et al., 2007; Ardilla Ardila and Ostrosky Solís, 2008; García-Molina et al., 2009). Luria (1966) proposed three complementary functional units: (1) the limbic system, in charge of motivation and warning; (2) posterior cortical areas (including visual, auditory, and general sensory regions), in charge of stimuli recollection, information processing and storage; and (3) the frontal cortex, in charge of executive functioning, control and monitoring of the activities we perform. Lezak (1982) defined executive functions as the mental components that enable goal-setting, planning strategies and the effective performance of actions. Baddeley (1996) grouped executive functions into cognitive processes for inhibition, cognitive flexibility, behavior planning and organization, and verbal fluency. The concept of executive functions comes mainly from neurological research on disorders suffered by persons with injuries to the brain cortex. Injuries to these lobes have a great impact on the life of these individuals, such as personality changes, difficulty with autonomy, and emotional and affective regulation (Baddeley and Wilson, 1988).

Baggetta and Alexander (2016) carried out a systematic review of 106 empirical studies where they established the common denominator of executive functions as cognitive processes that: (1) guide action and are essential to learning tasks and everyday actions; (2) help monitor these types of task; (3) control not only the cognitive but also the emotional and behavioral domains of human action. These authors report that the model most often adopted in research in that of Miyake et al. (2000), an integrating model with three executive components, distinct yet related to each other: (1) Updating information; (2) Inhibiting information; (3) Cognitive flexibility. Metcalfe and Mischel (1999) established two executive systems, referring to cognitive and emotional behaviors (Hongwanishkul et al., 2005; Brock et al., 2009; Prencipe et al., 2011; Arias and de los Ángeles, 2012, p. 44; Anderson and Bolden, 2018).

A recent work (Tirapu-Ustarroz et al., 2018) has analyzed, through a review of studies with exploratory and confirmatory factor analysis, the model models structural models underlying the existing empirical evidence. These authors conclude that the *executive functions models* evolve with age and incorporates planning factors in the later ages analyzed.

##### Neurocognitive treatment

Rubia (2018) carried out a review addressing the cognitive neuroscience of attention deficit hyperactivity disorder (ADHD). Their findings revealed functional brain abnormalities in ADHD subjects. Specifically, brain networks related to cognitive control, attention, timing, and working memory were impaired. In addition, distorted activity in brain regions that process motivation and emotion control and in the default mode network, was reported. On the other hand, it is interesting to note that, according to these abnormal activity patterns, classification of ADHD using functional magnetic resonance imaging (fMRI) data achieved satisfactory classification accuracies of over 80%.

In order to treat frontal functional impairment, transcranial direct current stimulation (tDCS) seems to be a promising tool in the improvement of ADHD symptoms and cognitive functions. However, larger clinical trials of repeated stimulation with and without cognitive training are needed to test clinical efficacy and potential costs in non-targeted brain functions. Regarding NF, its improvements in cognition and ADHD symptoms may be due to the placebo effect, since no differences in accuracy between experimental and control conditions have been found. Neurotherapeutics seems attractive for ADHD due to its safety and potential longer-term neuroplastic effects, which drugs cannot offer. However, short- and longer-term clinical and cognitive efficacy must be subjected to thorough testing, as well as the potential for individualized treatment.

This level of analysis, therefore, is optimal for explaining disorders based on neurological problems and brain injuries. There has been prolific evidence in recent years of the important role of the frontal cortex and the functional role of “executive functions” for predicting performance (Gathercole and Pickering, 2000a, b; Blair and Razza, 2007; Bull et al., 2008; Cleary and Chen, 2009; Agostino et al., 2010; Molfese et al., 2010; Monette et al., 2011; De los Ángeles, 2012; Diamond, 2012, 2013; Ramírez, 2014; Arán and López, 2016; Diamond and Ling, 2016; Cid-Sillero et al., 2018; Díez and Bausela, 2018; Follmer, 2018; Jacoby and Lavidor, 2018). An impairment in executive functioning has also been related to antisocial and delinquent behaviors (De Brito and Hodgind, 2009; Pera-Guardiola et al., 2016; Gil-Fenoy et al., 2018). Additionally, executive functions are associated with social competence and behavioral problems in preschool education (Romero-López et al., 2015), or in the delayed sleep-wake phase disorder (Wilhelmsen-Langeland et al., 2019).

##### Neuropsychological concept of “executive functions” and its limitations

“In the cognitive neuroscience literature, the term executive function is sometimes used interchangeably with self-control. The conceptual overlap between executive function and self-control is plain: core executive functions include top–down inhibitory control, working memory, and the cognitive flexibility to switch perspectives when demands require doing so” (El Haj and Allain, 2012; Gaillardin and Baudri, 2018; Duckworth et al., 2019, p. 376). Even though *executive functions* and their components (working memory, inhibitory control, and planning) have a proven neuropsychological contribution to self-regulation, and there is an established relationship between them (Diamond, 2013; Canet et al., 2016), we do not find a linear relationship between the conceptual models of research in the micro-, molecular-, and molar- analysis domains. Furthermore, despite this important evidence, models at the micro-analysis level overlook a large number of variables that *molecular* research has established in the explanation of behavior, such as the mediating role of personality, cognitive variables, learning strategies, learning styles, motivational style and coping strategies, not to mention the role of context (Meltzer, 2007; Best et al., 2011). For this reason, in the case of ADHD, the *neuropsychological* concept of “executive functions,” although adequate in its domain of micro-analysis, ought to be completed and connected to contributions from research at further levels of psychological research, mentioned above. Servera-Barceló (2005) comment “we express our desire that ADHD stop being a mere list of symptoms with a vague factorial support, to go on to incorporate those neurobehavioral characteristics that have received the support of the works empirical studies developed largely within the model of self-regulation” (p. 365).

#### Molecular Analysis Level (Behavioral “Software”): Self-Regulation

##### Definition and typology

*Self-Regulation* refers to both unconscious and conscious processes that affect the ability to control responses (Baumeister and Heatherton, 1996; Brown, 1998; Black and Allen, 2017; McClelland et al., 2018). This process has overarching effects on an individual’s ability to tolerate unmet wants or needs, handle disappointments and failures, and work toward success. The ability to self-regulate is the foundation for compliance with accepted standards of conduct at home, school, and later, in the workplace (vanDellen and Hoyle, 2008). This personal variable, therefore, has recently been considered a meta-skill or an ability to manage other skills (de la Fuente, 2017). Self-regulation is often thought of as:

(1) *Cognitive self-regulation* (meta-cognitive skills), the degree to which persons can be self-reflective, and can plan and think ahead. Persons with these strengths are in control of their thoughts. They monitor their behavior, evaluate their abilities, and are able to adjust their behavior, if necessary. For example, if a self-regulated youngster knows there is an upcoming test, he or she chooses to study to be ready for the test, instead of hanging out with friends.
(2) *Social-emotional self-regulation* (meta-emotional skills) is the ability to inhibit negative responses and delay gratification. An individual with this ability is able to control his or her emotional reactions to positive and negative situations, as in the case of a child who can resist his immediate inclination to erupt into anger when a peer cuts in front of him in the lunch line.
(3) *Behavior self-regulation* (meta-behavioral skills). Brown (1998, p. 62) saw self-regulation as an individual’s capacity to plan, monitor and direct his or her behavior in changing situations.”

Barkley’s (1998) model was initially based on behavioral inhibition, but eventually the limitations that were detected and new lines of research led him to advance toward self-regulation. The main components of the model are: behavioral inhibition processes, the very concept of self-regulation/self-control, the EF involved, and motor control. The model is applied to the behavior and characteristics of children with ADHD (for a model review, see Servera-Barceló, 2005). Complementarily, the Zimmerman model (Zimmerman and Labuhn, 2012; Panadero, 2017) of S*elf-Regulated Learning* has clearly shown that the self-control phase is the intermediate phase of self-regulation learning behavior. However, in the contexts of Personality or Clinical Psychology, Self-Control and Self-Regulation behavior are still considered equivalent, when the first is part of the second (Wiese et al., 2018; Duckworth et al., 2019).

Recent research has consistently reported that the *self-regulation* variable is a flourishing, positive, linear predictor of mental health, and a negative predictor of procrastination behaviors (Garzón-Umerenkova et al., 2018). In adolescents at risk for social exclusion, it is also a predictor of resilience (Artuch-Garde et al., 2017). It has further been established as a positive predictor of satisfaction in learning and performance (de la Fuente et al., 2015), as well as a negative predictor of anxiety (Ferrari et al., 2009; de la Fuente et al., 2017b) and the perception of maladaptive behaviors (de la Fuente et al., 2008; Ferrari et al., 2009). In complementary fashion, self-regulation has also been identified as an important procedural variable involved in adolescents’ competency in an interaction with alcohol, predicting low alcohol use (Ferrari et al., 2009; de la Fuente et al., 2017a). It has further been verified as a mediating variable in the effect produced by a mindfulness program (de la Fuente et al., 2018).

At the level applied to scholastic and academic learning, there is also an enormous amount of evidence regarding the importance and role of self-regulation during learning, i.e., self-regulated learning, in predicting motivation, learning and achievement (Schunk, 2005; Zimmerman and Labuhn, 2012; de la Fuente et al., 2014; Panadero and Alonso-Tapia, 2014; Greene et al., 2015, 2018; Bol et al., 2016; Sperling et al., 2016; Peters-Burton and Botov, 2017; Deekens et al., 2018; Greene, 2018; Li et al., 2018). In summary, the effects of self-regulation in scholastic and academic learning are (Schunk and Greene, 2018): (1) Higher academic achievement. Young people who are self-regulated are more likely to perform well in school; (2) School engagement. Adolescents who delay gratification and adjust their behavior are more likely to be engaged in school. Moreover, such students tend to work harder than their peers who lack self-regulating abilities do; (3) Peer social acceptance. Self-regulation is also linked to favorable perceptions by others. Children and adolescents who are able to control impulses and reflect on their actions are more likely to have friends and to get along with others; (4) Avoidance of negative behaviors. Self-regulated adolescents are less likely to engage in substance abuse, truancy, and violence; (5) Healthy eating patterns. Adolescents who are able to regulate their behavior are more likely to have healthy eating habits (Schutz and Davis, 2000; Strauman, 2002; Terry and Leary, 2011; Miller et al., 2018; Rademacher and Koglin, 2018).

##### Conclusion

Despite the important contributions of this variable, its theoretical domain does not allow for any accurate understanding of the effects of context, that is, of the educational environment, whether on academic behavior, or on behaviors related to health, work or social life. It is therefore necessary to include research at the next level in order to understand the interactive combination of *subject* × *context*.

#### Level of Molar Analysis (Real Interactive Behavior): Self- vs. External-Regulation

##### Definition

Despite abundant prior evidence, most models in use today whether produced at the level of micro-analysis or molecular analysis have paid little or no attention to the combined factors of self-regulation, that is, to the analysis of *personal factors* × *contextual factors.* Consequently, beginning with an awareness of the deeply interactive nature of behavior regulation, the theory of Self- vs. Externally-Regulated Behavior has been formulated at this level of psychological analysis (de la Fuente, 2017). See Tables 2, 3.

**TABLE 2:** Conceptual continuum and typologies of each self-regulatory behavior.

**Characteristics of the person**	**Self-regulation (SR) High-Moderate- Low POSITIVE PRO-ACTIVITY (+1)**	**A-regulation (AR) No regulation RE-ACTIVITY (0)**	**Dys-regulation (DR) Low-Moderate- High NEGATIVE PRO-ACTIVITY (−1)**
	**Before** Self-analysis of tasks Self-defined goals Self-motivation	**Before** No analysis of tasks No goals No motivation	**Before** Erroneous self-analysis Erroneous goals Self-demotivation
	**During** Self-observation Self-analysis Self-correction	**During** No self-observation No self-oversight No self-correction	**During** Self-distraction Cognitive self-avoidance Self-impediment Procrastination
	**After** Self-reflection Self-attributions Positive self-affect	**After** No reflection No attributions No affect	**After** Erroneous self-assessment Erroneous self-attributions Negative self-affect

**Type of activity**	**Self-regulatory (SR)** **High-Moderate-Low** **PRO-ACTIVITY** (+)	**A-regulatory (AR)** **No regulation** **RE-ACTIVITY** (=)	**Dys-regulatory (DR)** **Low-Moderate- High** **PRO-ACTIVITY (−)**

Academic	Self-regulated learning	No norms/limits	Self-induced impediment
Road safety	Self-regulation in driving	No norms/limits	Self-induced risks
Health	SR in Health	No norms/limits	Self-induced excesses
TV	SR in TV	No norms/limits	Self-induced excesses
Family	SR in family	No norms/limits	Self-induced risks
Information and Communication Technology (ICT)	SR in TIC	No norms/limits	Self-induced excesses
Sexual	SR in risky sexual behavior	No regulation	Self-induced risks
Violence	SR in harmonious relations	No norms/limits	Self-induced excesses
Spouse/partner	SR in interaction	No regulation	Self-induced excesses
			

**TABLE 3:** Conceptual continuum of the Externally-Regulated Learning (ERL) context dimension.

**Characteristics of the context**	**External self-regulation (ESR) High-Moderate-Low POSITIVE PROACTIVITY (+ 1)**	**External A-regulation (EAR) No regulation RE-ACTIVITY (0)**	**External dysregulation (EDR) Low-Moderate-High NEGATIVE PRO-ACTIVITY (−1)**
	**Before** Presents analysis of tasks Suggests adjusted goals Suggests self-motivation	**Before** Does not present tasks Does not propose goals Does not induce motivation	**Before** Erroneous tasks Erroneous goals (self-impediment) Induces demotivation
	**During** Promotes self-observation Promotes self-analysis Promotes self-correction	**During** No self-observation No self-oversight No self-correction	**During** Promotes self-distraction Cognitive self-avoidance, Self-impediment, Procrastination
	**After** Promotes self-reflection Promotes adjusted self-attributions Promotes positive adjusted self-affects	**After** No reflection No attributions No affects	**After** Promotes erroneous self-assessment, Erroneous self-attributions, Promotes maladjusted self-affects

**Type of Context**	**Externally-regulatory High Moderate Low**	**A-regulatory No regulation**	**Dysregulatory Low Moderate High**

Academic	Effective/regulatory teaching (RT)	Laissez-faire	Stressful teaching
Road safety	Correct traffic signs	No traffic signs	Road inducing speeding
Health	Norms/limits of consumption	No norms/consequences	Negative drinking contexts
TV	Norms/limits	No norms/limits	Negative TV contexts
Family	Authoritative/democratic	Permissive/laissez-faire	Liberal/promoting dysregulation
Information and Communication Technology (ICT)	Regulatory norms/limits	No norms/limits	Negative contexts
Sexual	Regulatory norms/consequences	No norms	Contexts which induce lack of control
Violence	Contexts with norms/values	No norms/values	Contexts which induce violence
Partner	Consensual interactions, norms in agreements	No norms	Changeable, unpredictable norms

This theoretical model postulates a combination of the subject’s level of self-regulation (SR), non-regulation (NR) or dysregulation (DR), with the context, which may be regulatory (RC), non-regulatory (NRC), or dysregulatory (DRC). This concept has the advantage of modeling the potential combinations of the regulatory level of the person and of the context, when explaining and predicting behaviors. Basically, the model can establish and predict the role of certain contexts by increasing or decreasing the likelihood of typical ADHD or attention deficit disorder (ADD) behaviors, assuming that these cannot be explained exclusively on the basis of neurological and individual characteristics. The model seeks to answer the following question: would the same person with low self-regulation (ADHD or ADD) behave the same way in a context that promotes self-regulation as they would in a context promoting dysregulation? The answer is that it seems unlikely. The model thus categorizes four possible effects resulting from the different combinations of levels of personal regulation (self-regulation) and a regulating context (promoting self-regulation). The proposed relationships have been tested with consistent effects in university teaching-learning processes (de la Fuente et al., 2015, 2017c), and are summarized in Table 4 below.

**TABLE 4:** Combination types between levels of variables in the Theory of Self- vs. Externally-Regulated Learning.

**Type**	**Presage**	**Process (deployment of teaching)**	**Process (deployment of learning)**	**Product**	
**Pintrich’s journey metaphor**	**Driver**	**Road**	**Driving conduct**	**Positive vs. negative emotions**	**Success vs. accident**

**Level**	**Personal self-regulation (SR) *(student)***	**Regulatory teaching (RT) *(context)***	**Self-regulated Learning (SRL) *(student)***	**Achievement emotions *(student)***	**Achievement behavior/cognitive *(student)***
4	High ⇒ low stress	High ⇒ low stress	High ⇒ deep approach Low ⇒ surface approach	High ⇒ + emotions Low ⇒ − emotions	High ⇒ academic achievement
3	High ⇒ low/medium stress	Low ⇒ medium/low stress	Medium/high ⇒ deep approach Medium/low ⇒ surface approach	Medium/high ⇒ + emotions Medium/low ⇒ − emotions	Medium/high ⇒ academic achievement
2	Low ⇒ medium/high stress	High ⇒ medium/high stress	Medium/low ⇒ deep approach High/medium > Surface approach	Moderate/Low ⇒ + emotions Moderate/High ⇒ − emotions	Moderate/low ⇒ academic achievement
1	Low ⇒ high stress	Low ⇒ high stress	Low ⇒ deep approach High ⇒ surface approach	Low ⇒ + emotions High ⇒ − emotions	Low ⇒ academic achievement

(1) Low personal self-regulation (personal *non-regulation/dysregulation*) with a non-regulatory context (*non-regulatory/dysregulatory context*). Think of a subject with ADHD/ADD, characterized by openness to experience associated risk behaviors, who lives in a family with divorced parents, receives contradictory instruction from them, lacks an organized schedule, has distracting daily activities and lacks externally-induced motivation for learning. What likelihood is there that medication alone can correct the problem? According to the proposed theoretical model, this combination would probably result in low achievement and a good number of socio-personal problems. This thesis would be consistent with results that find a significant correlation between ADHD and delinquent behaviors, substance abuse and personal failure (Holmes et al., 2019; Sadek, 2019) assigning it some degree of causality but without in depth analysis of any other explanatory variables, with unexplained latent effects in this association relationship.
(2) Low personal self-regulation (personal *non-regulation/dysregulation*) with a medium/high *regulatory* context. Think of a subject with ADHD/ADD characterized by openness to experience associated risk behaviors, who lives in a family with congruent parenting behaviors, with shared educational criteria and instruction, family organization in support of the child, a well-organized schedule, control over distracting activities, as well as externally-induced motivation and capacity for learning. What likelihood is there that an educational psychology intervention will enhance the effect of medication? This thesis would be consistent with evidence showing that the combined effects of medication and educational psychology intervention produce better effects, though without reaching an optimal level of achievement, learning, and development (Sherman et al., 2008; Dvorsky and Langberg, 2016).
(3) Medium/High *personal self-regulation* (SR) with a non-regulatory context. Think of a subject with ADHD/ADD with personal characteristics of medium/high regulation (SR), who lives in a family with divorced parents, receives contradictory instruction from them, lacks an organized schedule, has distracting daily activities and lacks externally-induced motivation for learning. What likelihood is there that self-regulated behavior will persist over time? According to the theoretical model, this combination would probably result in underachievement in comparison to the subject’s potential, and in certain socio-personal problems. This thesis would be consistent with evidence that refers to persons with ADHD having maladjusted behaviors as a consequence of a non-regulatory or dysregulatory context (Beattie, 2012). In this case, it is the context that is actively promoting dysregulation. We might consider that certain *false positives* fall into this case, children who are restless and lost, who have been socialized in a paradigm of dispersed attention, and are untrained in daily tasks, although they have potential for such training.
(4) Medium/High *personal self-regulation* (SR) with a medium/highly-regulatory context (RC). Think of a subject with a medium/high level of regulation, who lives in a family with congruent parenting behaviors, with shared educational criteria, family organization in support of the child, a well-organized schedule, control over distracting activities, mediation in external activities and friendships, as well as externally-induced motivation and capacity for learning. This thesis would be consistent with evidence that shows this to be best combination for optimal learning and academic achievement (de la Fuente et al., 2017c; Fong et al., 2018). In the sphere of developmental psychology, some recent studies have established the role of the family as an external regulatory context, and precursor of self-regulation and of executive function (Berner et al., 2010), consistent with postulates of the theoretical model presented here.

##### Conclusion

Introducing contextual variables into the study of self-regulatory processes seems to be essential, when we realize their role in determining behavior, whether in teaching processes (Vermunt, 1989; Vermunt, 2007; Timmons et al., 2016; Fryer and Vermunt, 2018), or in other contexts (Spruce and Bol, 2015).

### Assessment of Constructs

In Psychology, one of the parameters used for clearly defining a particular psychological construct has been the analysis of instruments associated with measuring that construct. This enables us to accurately understand the factors that make up the construct, as well as the theoretical-empirical adequacy of how the concept is formulated. Therefore, analyzing the different instruments in use should allow us to delimit the differences more accurately between the domains and/or levels of the constructs involved.

#### Assessment in Micro-Analysis: Executive Functions (see Supplementary Material Anex 1)

Esopo et al. (2018) state that “executive control is a broad term commonly referring to the maintenance and execution of high-level plans and goals, and involves planning, cognitive flexibility, inhibitory control, and working memory processes” (p. 31). They have summarized different inventories of executive functions. These include (see Supplementary  Material Anex 1).

#### Behavior Rating Inventory of Executive Function – Adult Version, BRIEF-A (Roth et al., 2005, 2013)

This self-report measure of executive function contains 75 items that assess attention regulation, inhibitory and emotion control, working memory, planning, and organization behaviors.

#### Consideration of Future Consequences (CFCs) (Strathman et al., 1994)

Consideration of Future Consequences quantifies the extent to which individuals consider potential future outcomes of their current behavior and is predictive of a number of health behaviors (Chapman, 2005), making it especially relevant for adherence. The CFC is a common, cross-culturally validated measure with attractive psychometric properties.

#### Deferment of Gratification Scale (DGS) (Ray and Najman, 1986)

As an alternative measure to the CFC, the Deferment of Gratification Scale (DGS) assesses the ability to resist the temptation of an immediate reward and instead wait for a larger, reward later (Carducci, 2009). The DGS comprises two factors relevant to adherence behavior: controlling impulses and planning and waiting. It was specifically designed to target intertemporal economic behavior, originally to explain social mobility or lack thereof. Participants choose ratings from “strongly disagree” (0) to “strongly agree” (5) on 12 items. Six items are ‘positive’ (such as, “I am good at saving my money”) and six are reversed (for example, “I agree with the philosophy ‘eat, drink and be merry, for tomorrow we may all be dead.”’).

Today, assessment of executive functions has evolved toward a virtual reality format, thereby improving its ecological validity and minimizing error factors (Díaz-Orueta et al., 2012, 2013; Bombín-González et al., 2014; Climent-Martínez et al., 2014; Azouvi et al., 2015; Iriarte et al., 2016; Nir-Hadad et al., 2017).

#### Assessment in Molecular Analysis: Self-Regulation (see Supplementary Material Anex 1)

##### Adolescent self-regulatory inventory

This is a 36-item questionnaire used to measure self-regulation of teens. Respondents rate how true each item is for them, ranging from 1 (not true of me at all) to 5 (quite true of me). A sum or average of the items is calculated. After reverse coding items 1, 2, 5, 6, 7, 8, 12, 13, 14, 15, 16, 18, 19, 21, 34, 35, higher scores indicate ability to self-regulate. The range of response is: (1) Not at all true of me; (2) Not very true of me; (3) Neither true nor untrue of me; (4) Somewhat true of me; (5) Quite true of me.

##### Self-regulation questionnaire (SRQ) (Brown et al., 1999)

This is a 63-item questionnaire used to measure self-regulation of teens. The questionnaire includes seven subscales that measure seven steps of self-regulation: receiving relevant information, evaluating the information and comparing it to norms, triggering change, searching for options, formulating a plan, implementing the plan, assessing the plan’s effectiveness. The short version has 17 items, for university students (Pichardo et al., 2014; Garzón-Umerenkova et al., 2017).

#### Assessment in Molar Analysis: Assessment of Self- vs. External-Regulation (see Supplementary Material Anex 1)

This level of assessment adopts the principles of interactivity by involving a joint assessment process, including both self-assessment and other-assessment of the teaching process and the learning process, under the premise that both of these will have either a regulatory nature (high score), an a-regulatory nature (mid-range score) or a dysregulatory nature (low score).

The *Interactive Assessment of the Teaching-Learning Process, IATLP* (de la Fuente and Martínez-Vicente, 2007), is based on the conception that self-regulation and *self-regulation* during the *learning process* may be induced externally, through *regulatory teaching* (de la Fuente and Justicia, 2003). The psychometric characteristics of this instrument are consistent and have been reported previously (de la Fuente et al., 2010).

## Discussion and Conclusion

The conceptual analysis offered here leads to several aspects worth mentioning for present and future research.

(1) We have observed how, at the micro-analysis level of basic psychological research (neuropsychological level), the construct most used is that of Executive Functions; at the molecular level of research, models use the construct Self-Regulation (or Self-Regulated learning), and at the molar level of research while maintaining the SR construct the context has also been incorporated in constructs, i.e., Self-Regulated vs. Externally-Regulated Learning. The concept of Executive Functions may be considered an essentially *psychoneurological or psychiatric* concept, that is, a medical concept, and therefore it minimizes the role of other personal and contextual variables, as well as being based on the assumption of the very concept of a “disorder” in connection with executive functions.
(2) It is clearly evident that some overlap exists between constructs and levels of psychological research, leading to confusion in the short term, and in the medium term fuzziness as to the proper connections between the different levels of research. If the *executive functions* construct is assumed to have certain elements in common with the *self-regulation* construct (SR), more behavioral in nature, and the similarities and differences are not clearly established, it may result in conceptual absorption and supplantation between different theoretical models that are complementary and belong to different research domains, as has been shown in this paper. A similar case has been shown between self-control and self-regulation, in which, in addition, it is intended to explain the performance in the absence of context variables or the teaching process (Duckworth et al., 2019) The impression is that each level of research and conceptual domain seeks to explain the totality of the problem, when this is only possible through integration and complementarity between the three levels of research.
(3) Adopting an approach from the exclusive perspective of micro-analysis while adequate for explaining neuropsychological disorders is plainly insufficient at the molecular level, and especially at the molar level, where contextual factors are essential for explaining behavioral variability and the *subject* × *context* interaction. In such significant behaviors as antisocial conduct, when basic research (micro-analysis level) takes a leap into psycho-educational or psychosocial implications (molar level), without considering possible *subject* × *context* interactions, it becomes too easy to conclude, risking attributional bias, that differences in executive functions in subjects with and without behavior or personality disorders explain these complex behaviors (for a review, see De Brito and Hodgind, 2009). While acknowledging the importance of contributions from micro-analysis, the fact that nothing else is known about the subjects’ characteristics or about their context must be recognized as an important limitation. If not, the socialization processes of these subjects, and what we know of their explanatory value (molecular level of psychological analysis), might be considered void, not to mention the influence of context in these behaviors (molar level of psychological analysis). From there, it would be easy to infer a deficit in executive functions as the cause of these maladjusted behaviors, disregarding any other factors (Pellerone et al., 2016). From a *neurological* and *clinical* point of view, this may be understandable (Anderson and Bolden, 2018), but from the psycho-educational and psychosocial viewpoint, it is simply unacceptable.

### Limitations, Implications, and Future Prospects

The essential limitation of this paper is that its focus is only analytical; it lacks an empirical meta-analysis of the publications produced at each level. This meta-analysis remains pending and it is necessary to do it in the future, to provide empirical evidence to the analyses made in this theoretical review. Nonetheless, we can infer certain implications from the foregoing analysis:

(1) Each level of Psychology research and analysis must *refocus* and *address* the level and research domain to which it belongs: micro-analysis, molecular analysis, or molar analysis. For this reason, new researchers must be taught the benefits and limitations of each level in question, so that they might find their place at their level of choice and perform their scientific work within their sphere of competence. In our case, we understand that while the construct of *executive functions* is attractive to researchers due to the halo effect coming from neurological support, the *self-regulation* construct possesses greater behavioral explanatory power (by incorporating the three levels of regulation, a-regulation, and dysregulation). Furthermore, it can (now) also incorporate the external component of regulation, through the construct of *external regulation* (regulatory, a-regulatory, and dysregulatory contexts), making it possible to carry out interactive, *subject* × *context* analyses, typical of Psychology’s domain as a behavioral science.
(2) It is essential that we work toward creating *cross-level research* that makes connections and validates the relationships between conceptual and empirical models at the different levels (Moore et al., 2018; Musso et al., 2019; Sánchez et al., 2019). Only in this way can we verify the external validity of each construct, not only between constructs at the same level, but also between constructs in different research levels and domains. Some prior studies have already begun to investigate in this direction, connecting *executive functions –* > *self-regulation –* > *external regulation* (Calkins and Marcovitch, 2010; Follmer and Sperling, 2016, 2018a,b,c; Aadland et al., 2018; Baumeister et al., 2018; Rutherford et al., 2018; Wagner et al., 2018; Wimmer et al., 2019). However, studies are needed that evaluate the relevance and potential role of context in self-regulating behavior, and its role as a regulatory/non-regulatory/dysregulatory agent, all of this in different behavioral issues, as established by SRL vs. ERL theory. Lacking this, just as we speak of *neuromyths in education* (Howard-Jones, 2014), alluding to erroneous educational conceptions about the brain and its functioning, unsupported by neurological science, we might also speak of *psychomyths in neurology*, alluding to the myths of neurology that psycho-educational science does not support at molecular and molar levels. The very concept of *neuro-education* attempts to establish a connection between the two distal levels, micro-analysis/neurology and molar analysis/education (for a detailed analysis, see Pallarés-Dominguez, 2016), without regard for the qualitative leap and the difficulty involved in connecting and conceptually integrating the two levels of research. “*Executive functioning* lays the basis for *behavioral self-regulation* in real-life contexts, such as adapting one’s behavior according to social standards and working consistently toward long-term goals despite the temptations of short-term pleasures (it is important to note that several terms with overlapping definitions, such as behavioral self-regulation, effortful control, and self-control, are used in the literature with regard to the construct we are referring to as behavioral self-regulation)” (Gunzenhauser et al., 2017, pp 30–31).
(3) *Research ethics* must be kept in mind, reminding us that publications that introduce and promote psychological constructs must be unequivocally backed by evidence presented in empirical research. If this is not the case, opinion trends and new myths will be created, based on a false belief in the unlimited explanatory power of Neurology and Psychiatry, to the detriment of important, consolidated scientific contributions from other levels of Psychology research.

## Author Contributions

JF have made the general analysis and first version of the manuscript. MG-T has made the second review of the Self-regulation and Externally regulation sections of ADHD. MA-S has performed a review of the neuroanalysis level of ADHD. JM-V has carried out the carried out the revision bibliographic by sections. FP-S has reviewed the professional applicability of the manuscript. MV has carried out a review of the section on professional ethics and future vision of research.

## Conflict of Interest Statement

The authors declare that the research was conducted in the absence of any commercial or financial relationships that could be construed as a potential conflict of interest.
