# Supplementary material for: Implications of Unconnected Micro, Molecular, and Molar Level Research in Psychology: The Case of Executive Functions, Self-Regulation, and External Regulation
Source: Front Psychol. 2019 Aug 27;10:1919. doi: 10.3389/fpsyg.2019.01919 (PMC6719524; doi:10.3389/fpsyg.2019.01919)
Supplement: Supplementary file 1 [file Table_1.DOCX]

**ANEX. ASSESSMENT (COMPLEMENTARY MATERIAL)**

**Examples of Assessment at the microanalysis level: Executive Functions**

*Behavior Rating Inventory of Executive Function - Adult Version* (BRIEF-A)

1. I do not have problems completing my work

2. I make careless mistakes when completing work

3. I have trouble being attentive while working (such as household chores, reading or work).

4. I need to be reminded to start a task even when it's my own will.

5. I get overwhelmed by large tasks.

6. I have trouble with jobs or tasks that have more than one step.

7. I have trouble getting ready for the day.

8. When I have many important things to do, I have trouble deciding which activity to start ﬁrst.

9. I forget what I am doing in the middle of things/activities.

10. I don't inspect my work for mistakes.

11. I lay around in the house a lot.

12. I start work (such as cooking, projects) without the right tools.

13. I fail to judge how diﬃcult or easy work will be.

14. I have trouble starting anything on my own.

15. I have trouble staying on the same topic when talking.

16. I don't plan early for future activities.

17. I concentrate for a short time.

18. I have goals that are unachieveable.

19. I make mistakes carelessly.

20. I have diﬃculty being excited about things.K.

21. I forget instructions easily.

22. I have good ideas but cannot put my ideas into action.

23. I have trouble getting started on tasks.

24. I have trouble ﬁnishing tasks (such as chores, work).

25. I start things at the last minute (such as assignments, chores, tasks).

26. I have diﬃculty ﬁnishing a task on my own.

27. I have trouble remembering things, even for a few minutes (such as directions, phone numbers).

28. I have trouble coming up with ideas for what to do with my free time.

29. I don't plan early for tasks.

30. I have problems organizing activities.

31. I have trouble doing more than one thing at a time.

32. I have trouble organizing work.

34. I do not make careless mistakes when completing my work.

*Consideration of Future Consequences (CFC)*

1. I think about how things would be in days to come, and try to inﬂuence those things in my daily behavior.

2. I often involve myself in a speciﬁc behavior to achieve results that may not come until many years later.

3. I only act to satisfy immediate needs, thinking the future will take care of itself.

4. My behavior is inﬂ uenced by the immediate outcomes of my actions (i.e. within a few days or weeks).

5. When I take action or make decisions, I am more likely to choose an option that involves little trouble or eﬀort.

6. I am ready to sacriﬁce my current happiness or wellbeing in order to achieve future results.

7. I think it's important to take warnings on bad outcomes as a very weighted issue even if the bad outcome may not happen until many yearslater.

8. I think it is important to work on something with important future consequences than on something with less important immediateconsequences.

9. In general, I ignore warnings about problems that can possibly happen later because I think those problems will be solved before reachinga crisis level.

10. I think that sacriﬁcing now is not a must because later outcomes can be dealt with at a later time.

11. I only act to satisfy immediate concerns, ﬁguring that I will take care of the future problems that may occur at later date.

12. Since my everyday work has speciﬁc outcomes, it is more important to me than behavior that has distant outcomes.

13. When I make a decision, I think about how it might aﬀect me in the future.

14. My behavior is generally inﬂuenced by future consequences.

*Deferment of Gratiﬁcation (DGS)*

1. I am good in saving my money instead of spending it at once.

2. I enjoy something more when I have to wait for it and plan for it.

3. When I was a child, I saved any pocket money that I had.

4. When I am in the market, I usually buy a lot of things that I had not planned to buy.

5. I am constantly without money.

6. I agree with the philosophy: “Eat, drink, and be happy, for tomorrow we may all be dead”.

7. I would describe myself as often acting without thinking for my own good.

8. I often think it is important to wait and think things over before deciding.

9. I like spending my money immediately after I get it.

10. It is hard for me to avoid losing my temper when someone gets me very angry.

11. Most of the time, it is easy for me to be patient when I am kept waiting for things.

12. I am good at planning things ahead.

**Examples of Assessment in the molecular level: Self-Regulation**

*Adolescent Self-Regulatory Inventory* (Moilanen, 2007).

1. It’s hard for me to notice when I’ve had enough (sweets, food, etc.).

2. When I’m sad, I can usually start doing something that will make me feel better.

3. If something isn’t going according to my plans, I change my actions to try and reach my goal.

4. I can find ways to make myself study even when my friends want to go out.

5. I lose track of the time when I’m doing something fun.

6. When I’m bored I fidget or can’t sit still.

7. It’s hard for me to get started on big projects that require planning in advance.

8. I can usually act normal around everybody if I’m upset with someone.

9. I am good at keeping track of lots of things going on around me, even when I’m feeling stressed.

10. When I’m having a tough day, I stop myself from whining about it to my family or

friends.

11. I can start a new task even if I’m already tired.

12. I lose control whenever I don’t get my way.

13. Little problems detract me from my long-term plans.

14. I forget about whatever else I need to do when I’m doing something really fun.

15. If I really want something, I have to have it right away.

16. During a dull class, I have trouble forcing myself to start paying attention.

17. After I’m interrupted or distracted, I can easily continue working where I left off.

18. If there are other things going on around me, I find it hard to keep my attention focused

on whatever I’m doing.

19. I never know how much more work I have to do.

20. When I have a serious disagreement with someone, I can talk calmly about it without

losing control.

21. It’s hard to start making plans to deal with a big project or problem, especially when I’m feeling stressed.

22. I can calm myself down when I’m excited or all wound up.

23. I can stay focused on my work even when it’s dull.

24. I usually know when I’m going to start crying.

25. I can stop myself from doing things like throwing objects when I’m mad.

26. I work carefully when I know something will be tricky.

27. I am usually aware of my feelings before I let them out.

28. In class, I can concentrate on my work even if my friends are talking.

29. When I’m excited about reaching a goal (e.g., getting my driver’s license, going to

college), it’s easy to start working toward it.

30. I can find a way to stick with my plans and goals, even when it’s tough.

31. When I have a big project, I can keep working on it.

32. I can usually tell when I’m getting tired or frustrated.

33. I get carried away emotionally when I get excited about something.

34. I have trouble getting excited about something that’s really special when I’m tired.

35. It’s hard for me to keep focused on something I find unpleasant or upsetting.

36. I can resist doing something when I know I shouldn’t do it.

*Self-Regulation Questionnaire. SRQ* (Brown, Miller & Lendonsky,1998; Short version: Pichardo, et al, 2014):

*Goal setting*

42. I set goals for myself and keep track of my progress.

47. Once I have a goal, I can usually plan how to reach it.

49. If I make a resolution to change something, I pay a lot of attention to how I’m doing.

33. I have a hard time setting goals for myself.

1. I usually keep track of my progress toward my goals.

40. I have trouble making plans to help me reach my goals.

*Perseverance*

34. I have a lot of willpower.

6. I get easily distracted from my plans.

41. I am able to resist temptation.

*Decision making*

5. I have trouble making up my mind about things.

12. I put off making decisions.

19. When it comes to deciding about a change, I feel overwhelmed by the choice.

55 Litle problems or distractions throw me off course.

13. I have so many plans that it’s hard for me to focus on any one of them.

*Learning from mistakes*

21. I don’t seem to learn from my mistakes.

28. I usually only have to make a mistake one time in order to learn from it.

57. I learn from my mistakes.

**Exmaples of Assessment in the molar level: Self vs External Regulation**

*Interative Assessment of the Teaching Learning Process, IATLP Scale* (de la Fuente & Martínez-Vicente, 2007)

*Self- Regulated Learning*

Part A. *Self-Regulated Learning Behavior in the Classrrom*

1. At the beginning of each topic or lesson, I want to know why we are going to learn this material.

2. At the beginning of each activity, I think about why we are going to do it.

3. At the beginning of each topic or lesson, l map out the material we are going to work on using either a conceptual map (graphic which shows relationships between concepts), a diagram, outline, script, or other.

4. At the beginning of each topic or lesson, I think about relationships that exist between material we are going to work on, and other material learned previously.

5. For each topic or lesson, I know the different types of subject matter we are going to work with: facts and concepts, procedures, or attitudes, values and norms.

6. For each topic or lesson, I know which material is most important to learn.

7. When doing learning activities, I talk with my classmates about how they should be done.

8. I ask questions, and don't just keep my doubts to myself.

9. I realize when I have problems learning, and ask for help.

10. I give myself a certain amount of time for doing activities that are assigned.

11. I make a work plan for each topic or lesson.

12. I use some activity (oral questions, questionnaire, etc.) to evaluate what I already know when beginning an instructional unit.

13. I use some activity (test, questionnaire, oral questions, personal journal, etc.) to evaluate what I have learned at the end of the topic or lesson.

*Part B. Learning and Self-Regulation Strategies*

14. I try to learn things by repeating them aloud while I am reading.

15. When I'm trying to memorize something, I usually use memory devices, making associations with words or ideas.

16. I usually write things down in order to memorize them.

17. In order to remember what I'm learning, I usually use a guide, dividing the topic into parts, and dividing up the parts into other smaller ones.

18. Before learning new material, I usually skim through the topic to see what it's about. 19. I usually underline ideas or words that I want to stand out in the text.

20. I usually draw out the most important ideas from the topic I am learning about and write them down.

21. I usually write in headings or subheadings for paragraphs or chunks of text that I am learning from.

22. I usually write summaries about what I am learning.

23. I usually make notations, either in the book or in a notebook, when learning new material. A B C D E 24 For each topic or lesson, I usually ask myself and try to discover what the central idea is. A B C D E 25 When learning, I usually differentiate between important ideas and details.

26. When I am learning and I encounter some difficulty, I go on even though I don't understand.

27. When learning, I focus more on the words, terms, or the data rather than on discovering the meaning of the text.

28. I usually make charts or outlines that represent everything I've learned.

29. When learning, I usually make graphic representations or drawings of what I am studying.

30. I like to express in my own words the meaning of paragraphs that I am studying.

31. When I am about to learn new material, I try to ask myself questions about what I'm going to read.

32. When learning new material, I try to relate it to other knowledge I already have, looking for similarities and differences.

33. When learning, I like to relate it to my own experience and my life.

34. When learning about something, I try to put it into practice or apply it to reality, whether present or future.

35. When learning, I try to think of mental images that will help me intensify my learning experience.

36. When learning, I try to broaden what I learn by consulting different books or media. 37. When learning about something, I like to think about it and ask myself questions, using with my own reflections and considerations.

38. Before beginning any activity or learning task, I usually take into account what I need to know, and how much work and time I must devote to it.

39. Before beginning any activity or learning task, I usually divide the task or activity into parts, to make it easier for me.

40. Before beginning any activity or learning task, I organize what I have to do, telling myself: "first I have to do this, then I have to do that ...”

41. Before beginning any activity or learning task, I usually look ahead, calculating the time I have available in order to realistically distribute it among the aspects or elements involved.

42. Before beginning any activity or learning task, I usually take into account the different materials, books or documents I'm going to need.

43. Before beginning any activity or learning task, I try to find the best conditions of place, time and companions, so that these elements do not produce distractions.

44. When carrying out an activity or learning task, if there is something I don't understand or I don't know what to do, I try to keep going until I can resolve it.

45. When carrying out an activity or learning task, if some problem or question comes up, I always try to find the solution myself.

46. When carrying out an activity or learning task, when I can't manage to solve a problem myself, I turn to others for help.

47. When carrying out an activity or learning task, I try to check the opinions of others to see if what I'm doing is correct.

48. When I have finished an activity, I have the habit of checking everything to see if I've made any mistakes.

49. When I have finished an activity, I try to relate what I've done with what the teacher has asked for.

50. I keep my attention on the activity I am doing, and don't get distracted.

51. Before starting an activity, I tend to think that I can do a good job.

52. I think that if I make an effort, generally speaking, I'm able to learn what I want to. 53. When I don't feel like doing an activity, I usually start with the easiest or most attractive part in order to motivate myself.

54. Before starting an activity, I try to consider the importance, the interest or the usefulness of what I'm going to do.

55. Before starting an activity, I tend to give myself challenges in order to get motivated, such as: "I'm going to learn this in half an hour ", "today I'm going to go as far as page X".

56. I usually take breaks when working on activities or assignments.

57. I change activities often in order to keep interested in what the class is learning about. 58 I feel satisfied when I've learned something new.

*Regulatory Teaching (External regulation)*

*Part A. Teacher’s General Behavior*

1. At the beginning of each unit or lesson, the teacher explains why we are going to learn the material.

2. At the beginning of each activity, the teacher explains why we are going to do it.

3. The teacher explains the objectives of the activities we are going to carry out.

4. The teacher tries to determine whether the students have understood the learning objectives well.

5. The teacher presents the subject matter we are going to work on using some kind of conceptual map, diagram, chart, script, etc.

6. The teacher shows existing relationships between the material we are going to work on and other material we learned previously.

7. The teacher indicates which content items are the most important ones to learn in each unit or lesson.

8. The teacher makes the classes enjoyable.

9. The teacher is concerned that students feel comfortable in class.

10. The teacher is clear and orderly in his or her explanations.

11. The teacher frequently informs us as to our progress in the subject.

12. The teacher allows us to speak in class about how we are learning.

13. The teacher takes time to address our concerns or doubts.

14. The teacher helps us with corrections.

15. The teacher realizes when students have trouble learning a topic.

16. The teacher explains how we are going to be evaluated.

17. The teacher allows participation in evaluating his teaching.

18. The teachers makes us reflect on our learning in order to improve it.

*Part C. Regulation Activities in Learning*

30. The teacher does learning preparation activities with us.

31. The teacher presents a class work plan for each lesson or topic.

32. The teacher negotiates with the students an individual work plan for the whole lesson or topic.

33. The teacher uses some activity (dialogue, questionnaire, etc.) to evaluate what we know when beginning a lesson or topic.

34. The teacher uses some activity (dialogue, questionnaire, etc.) to evaluate what we know while the lesson or topic is in progress.

35. The teacher uses some activity (dialogue, questionnaire, etc.) to evaluate what we know when we have finished the lesson or topic.

36. While we are learning, the teacher dialogues with the students about the objectives of the lesson or topic.

37. While we are learning, the teacher creates opportunities so we can think together about how we are learning.

38. While we are learning, the teacher helps us to make clear and realistic learning goals. 39. While we are learning, the teacher works with us on skills for reviewing and modifying our learning objectives.

40. While we are learning, the teacher makes us think about the way we are learning: at the beginning, during and after finishing the activities.

41. While we are learning, the teacher asks us to reflect, instead of making us repeat all the information.

42. While we are learning, the teacher makes us feel satisfied through learning “how to learn better”.
